# Head and Cervical Posture in Sagittal Skeletal Malocclusions: Insights from a Systematic Review

**DOI:** 10.3390/jcm14082626

**Published:** 2025-04-11

**Authors:** Gianna Dipalma, Alessio Danilo Inchingolo, Carmela Pezzolla, Roberta Sardano, Irma Trilli, Daniela Di Venere, Massimo Corsalini, Francesco Inchingolo, Marco Severino, Andrea Palermo, Angelo Michele Inchingolo

**Affiliations:** 1 Department of Interdisciplinary Medicine, University of Bari “Aldo Moro”, 70124 Bari, Italy; giannadipalma@tiscali.it (G.D.); alessiodanilo.inchingolo@uniba.it (A.D.I.); carmela.pezzolla@uniba.it (C.P.); roberta.sardano@uniba.it (R.S.); trilliirma@gmail.com (I.T.); daniela.divenere@uniba.it (D.D.V.); massimo.corsalini@uniba.it (M.C.); angeloinchingolo@gmail.com (A.M.I.); 2 Department of Medicine and Surgery University of Perugia, 06132 Perugia, Italy; marco.severino@unipg.it; 3 Department of Interdisciplinary Medicine, University of Salento, 73100 Lecce, Italy; andrea.palermo@unisalento.it

**Keywords:** biomechanics, cervical spine, cervical vertebrae, cranial bones, malocclusion, neck, orthodontics, posture, skeletal malformations

## Abstract

**Background:** This systematic review aims to evaluate the relationship between craniocervical posture and sagittal skeletal malocclusions, focusing on cervical curvature, head posture, and the influence of skeletal classification on craniofacial development. **Methods:** A comprehensive electronic search was conducted across PubMed, Scopus, and Web of Science for studies published between January 2015 and January 2025. Studies meeting the PICOS criteria, which assessed craniocervical posture in individuals with skeletal Class I, II, or III malocclusions, were included. A total of 12 studies were reviewed and analyzed for relevant data. **Results**: Significant correlations were identified between sagittal skeletal malocclusions and craniocervical posture, particularly cervical curvature. Class II malocclusion was associated with increased cervical curvature and forward head posture, whereas Class III malocclusion was linked to straighter cervical columns and a more posterior head position. Variations in cervical vertebral morphology were also observed, especially in relation to head posture and craniofacial structure. However, considerable heterogeneity was noted among studies regarding sample populations, measurement techniques, and classification criteria. **Conclusions:** This review highlights a strong interrelationship between craniocervical posture and sagittal skeletal classification, with potential clinical implications for orthodontic diagnosis and treatment planning. Further longitudinal studies are needed to establish causal relationships and improve orthodontic management strategies.

## 1. Introduction

Poor head and neck posture is considered one of the primary causes of myofunctional disorders in the craniofacial region [1,2,3,4,5]. Abnormal head and neck posture during a patient’s active growth phase may disrupt the normal development of craniofacial structures due to the biomechanical and anatomical connection between neck muscles and craniofacial structures. The positioning of the head and neck is closely linked to the alignment and development of the jaw and the maxillofacial skeleton [6,7,8,9]. When the head is improperly aligned with the cervical spine, it can place abnormal stresses on the temporomandibular joint (TMJ) and muscles responsible for mastication and facial expression [10,11,12,13,14,15,16]. In fact, changes in the quadrilateral area affect the asymmetry of the chewing muscles [17]. This imbalance may lead to malocclusion, which refers to the misalignment of the teeth or jaws. The craniofacial structures and cervical spine share a dynamic relationship, and abnormal posture can influence the function and development of both systems, resulting in long-term functional and aesthetic consequences for individuals [18,19,20,21,22,23,24].

**Figure 1:**
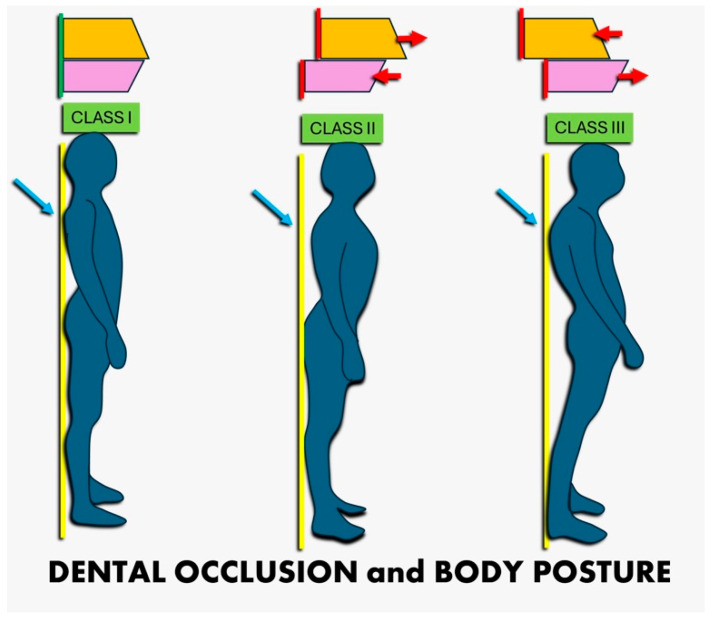
Correlation between cervical posture and malocclusion. The image illustrates the relationship between cervical spine alignment and dental malocclusion, highlighting how changes in cervical posture can influence occlusal balance and craniofacial dynamics. In particular, if the jaw is retruded (as it often is in Class II), the body might compensate by tilting the head forward to facilitate breathing or swallowing, while in subjects with Class III, the prominent jaw may induce a more flexed (i.e., forward tilted) head posture to balance occlusion and horizontal vision.

Moreover, previous epidemiological studies have indicated that individuals with neck disorders show a higher prevalence of craniomandibular disorders (CMDs), which are frequently linked to malocclusion (Figure 1). CMDs encompass a wide range of conditions affecting the temporomandibular joint and the muscles responsible for jaw movement [25,26,27,28,29]. These disorders can cause symptoms such as jaw pain, difficulty in mouth opening or closing, and facial discomfort, often exacerbated by poor head and neck posture [30,31,32,33,34,35,36,37,38,39,40,41,42,43]. Epidemiological studies have shown that patients with tinnitus often exhibit symptoms consistent with CMD. A study involving 102 tinnitus patients found that CMD symptoms, such as frequent headaches, jaw muscle tenderness, and impaired mandibular mobility, were significantly more prevalent in these patients compared to general populations. This finding suggests that CMD may contribute to the severity of tinnitus in some individuals [44]. The concurrent presence of CMDs and malocclusion in patients with neck disorders suggests a notable association, although the exact nature of this relationship warrants further investigation [45,46,47,48].

**Figure 2:**
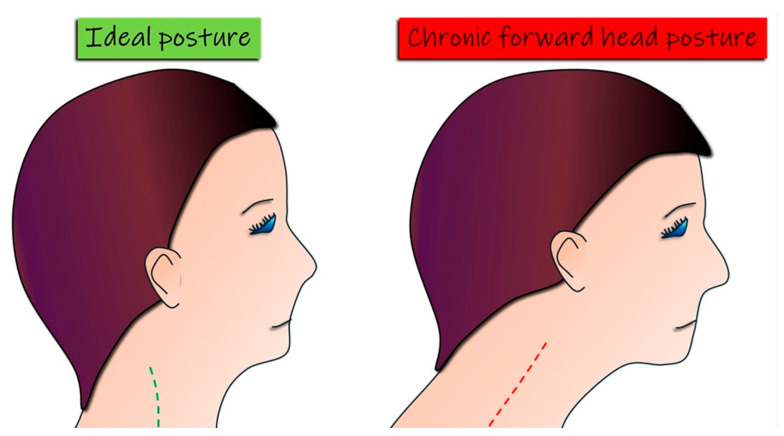
Ideal posture vs. chronic forward head posture. The image compares ideal cervical alignment with chronic forward head posture, showing how forward displacement of the head can affect spinal alignment, muscle balance, and overall postural stability.

A fundamental hypothesis in orthodontics and orthopedics is that head and cervical posture are directly related to malocclusion [49,50]. Studies suggest that cervical spine alignment affects mandibular positioning and the overall facial skeletal structure [51,52,53,54,55]. For instance, recent evidence indicates that children with Class II malocclusion—characterized by a convex profile with a retrognathic mandible and/or a prognathic maxilla—tend to exhibit greater head extension relative to the spinal column. This altered posture often results in a more posterior head position concerning the body, potentially impacting craniofacial development [56,57,58,59,60]. Conversely, individuals with Class III malocclusion—characterized by a concave profile with a prognathic mandible and/or a retruded maxilla—exhibit a significantly reduced cervical lordosis angle [61,62,63,64,65]. A decrease in cervical lordosis, the natural inward curvature of the cervical spine, may represent a compensatory adaptation to sustain an upright head posture, potentially affecting jaw and facial alignment [66,67,68,69,70,71]. (Figure 2).

Building on these findings, several conservative and physical therapies have been developed to correct abnormal head and cervical posture [72]. Techniques such as continuous postural monitoring, transcutaneous electrical nerve stimulation (TENS), bracing, and smart posture corrective orthoses have shown promising potential in preventing the onset of sagittal malocclusion. These therapies, when implemented early in life or at the onset of craniofacial development, may serve as preventative measures to mitigate the risk of developing sagittal malocclusion [73,74,75,76,77,78].

Despite encouraging findings from various studies, the hypothesis connecting head and cervical posture to malocclusion remains debated among researchers [79,80,81]. A key criticism is that many existing studies do not adequately control for potential confounding factors, such as age, which can affect the degree of cervical lordosis [82,83,84,85,86,87,88,89]. Natural age-related changes in the spine can lead to alterations in head and neck posture, which may, in turn, influence the development of malocclusion [90,91,92,93,94,95,96,97]. Additionally, abnormal jaw relationships could be attributed to developmental disorders rather than cervical posture alone. This relationship holds significant implications for both orthodontic and orthopedic fields, as it could guide the development of new treatment protocols and preventive strategies aimed at improving craniofacial development [98,99,100,101,102,103,104,105,106]. Therefore, a deeper understanding of the impact of age on the relationship between cervical posture and malocclusion is crucial. Further longitudinal studies that control for age and other potential confounders are needed to provide more definitive evidence [107,108,109].

## 2. Materials and Methods

### 2.1. Methodology

This systematic review was conducted in full compliance with the PRISMA (Preferred Reporting Items for Systematic Reviews and Meta-Analyses) guidelines, ensuring transparency and rigor in the review process [110]. The protocol for the review was registered with PROSPERO under CRD420251008415.

The rationale for this review was to address the existing gap in the literature regarding the relationship between craniocervical posture and sagittal skeletal malocclusions, which remains an underexplored topic. The PRISMA checklist was followed throughout the review process to ensure full transparency and to reduce bias, addressing key aspects such as the search strategy, data synthesis, and methodological quality assessment [111].

### 2.2. Research Processing

An electronic search was performed in PubMed, Scopus, and Web of Science to identify studies published in English between 1 January 2005 and 1 January 2025. The search strategy included combinations of keywords such as “cranio-cervical posture”, “malocclusion”, “Class II malocclusion”, “Class III malocclusion”, “head posture”, and “cervical spine alignment”, combined with Boolean operators like AND, OR, and NOT. Specifically, the terms “cranio-cervical posture” AND “malocclusion” were used to retrieve studies linking both concepts, while “Class II malocclusion” OR “Class III malocclusion” was used to capture studies examining different types of malocclusions. Boolean operators were applied to ensure comprehensive coverage of various aspects of craniocervical posture and sagittal skeletal malocclusions. The inclusion of only English-language studies was based on practical considerations, such as language proficiency and access to reliable translation services; however, this decision may introduce potential language bias.

### 2.3. Eligibility Criteria

Studies were selected based on the PECOS framework (Table 1):

- Population (P): Individuals with skeletal Class I, II, or III malocclusions, diagnosed through cephalometric analysis.
- Exposure (E): Craniocervical posture assessed through cephalometric measurements and cervical curvature analysis.
- Comparison (C): Differences in postural parameters between individuals with different skeletal classifications or comparisons with normocclusal subjects.
- Outcome (O): Association between craniocervical cephalometric parameters and sagittal skeletal classification, with a focus on cervical angles and head position.
- Study Design (S): Observational studies (cross-sectional, case–control, and longitudinal) evaluating craniocervical posture in relation to sagittal malocclusion.

This structured framework was selected to ensure a comprehensive review of studies that directly assess craniocervical posture in relation to malocclusion types, including only studies using objective and reliable methods, such as cephalometric analysis, to minimize bias.

### 2.4. Exclusion Criteria

The exclusion criteria were as follows:

- Case reports: Studies that describe individual or a small number of cases, without broader applicability to the research question, were excluded.
- Animal studies: Only human studies were included to ensure relevance to the clinical context.
- In vitro studies: Studies conducted in laboratory settings without human participants were excluded.
- Other reviews: Systematic reviews, meta-analyses, and narrative reviews were excluded to avoid redundancy and to focus on primary research articles that offer novel data on the relationship between craniocervical posture and sagittal skeletal malocclusions.
- Off-topic studies: Studies not directly related to the relationship between craniocervical posture and sagittal malocclusions were excluded. For example, studies focusing solely on the effects of orthodontic treatments unrelated to posture, or those dealing with other health conditions unrelated to skeletal malocclusions, were not included. This criterion ensured that the review remained focused on the core research question.

### 2.5. Data Extraction and Analysis

Data extraction was performed by two independent reviewers. Discrepancies between reviewers were resolved through discussion, and if necessary, a third reviewer was involved to reach a consensus. The process of resolving disagreements was closely monitored to ensure the consistency of data extraction across studies.

No formal calibration process was conducted prior to data extraction. However, the data extraction process was carefully coordinated, and any discrepancies in data interpretation were resolved promptly by the team.

The methodological quality of the included studies was assessed using the Newcastle–Ottawa Scale (NOS). This scale was applied to evaluate the quality of observational studies, considering factors such as the selection of participants, the comparability of groups, and the assessment of outcomes. The results from the quality assessment were used to inform the analysis, helping to identify potential sources of bias and methodological limitations in the studies.

Primary outcome measures included cephalometric angles such as NSL-Ver, NL-Ver, ML-Ver, OPT/HOR, MCA, SN-Ver, SN/CVT, and OPT/CVT. These measurements were selected due to their direct relevance to the study objectives, which aimed to assess the relationship between craniocervical posture and sagittal skeletal classification.

A qualitative synthesis of the findings was performed to compare the available evidence. The data were analyzed to explore common patterns, trends, and discrepancies across studies. Heterogeneity across studies was considered and discussed, particularly in relation to variations in study design and measurement techniques. The review aimed to provide a comprehensive overview of the current literature and identify areas where further investigation is needed.

## 3. Results

A total of 18,976 publications were identified through online databases: PubMed (n = 13,863), Scopus (n = 3496), and Web Of Science (n = 1617). No additional studies were identified through manual research. After removing 13,657 duplicate records, 5319 studies remained and were screened by title and abstract.

**Figure 3:**
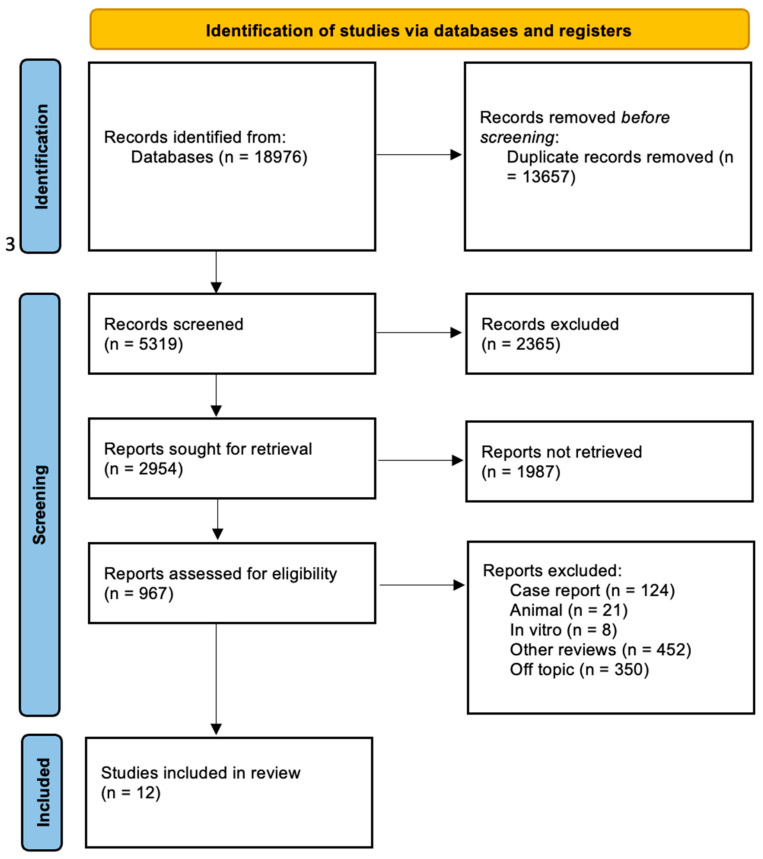
PRISMA flow diagram.

Following a detailed eligibility assessment of the 867 reports, 855 were excluded for not meeting the criteria (Figure 3). The selection process and summary of included records are illustrated in Table 2.

### Quality Assessment and Risk of Bias of Included Articles

**Figure 4:**
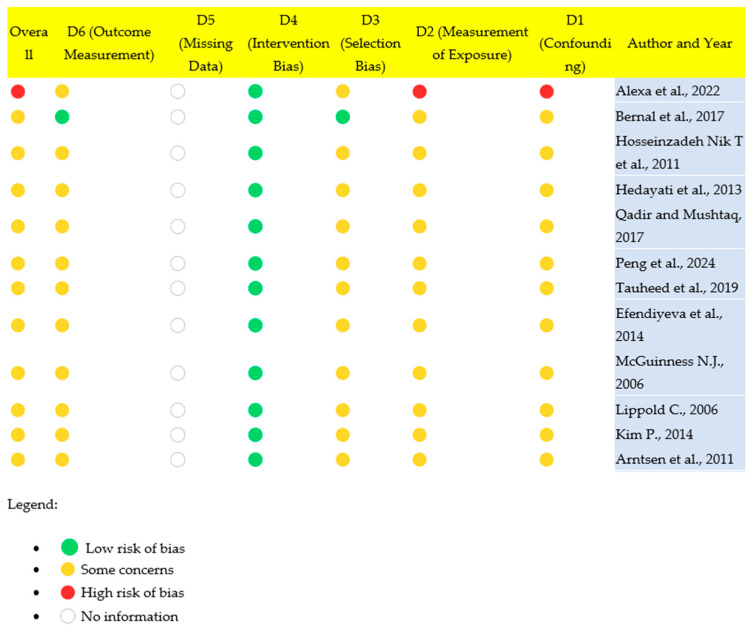
Representation of the risk of bias [9,24,26,76,112,113,114,115,116,117,118,119].

The methodological quality of the included studies was assessed using the Newcastle–Ottawa Scale (NOS). The cross-sectional studies were evaluated using the NOS adaptation for cross-sectional studies, while for any cohort or case–control studies, the corresponding NOS versions were applied. The studies included in the review generally present a moderate risk of bias, with several factors contributing to this evaluation. The studies included in the review generally present a moderate risk of bias, with several factors contributing to this evaluation. For instance, Alexa et al. (2022), which is a retrospective observational study, carries a high risk of bias due to its reliance on existing data from lateral cephalograms, limiting control over data collection and increasing susceptibility to confounding variables [9]. The Bernal et al. (2017) study, a cross-sectional study, shows some concerns due to its use of a relatively small sample size and potential selection biases, as the findings are derived from a specific age group of children [118]. Similarly, studies like Hedayati et al. (2013) and Qadir and Mushtaq (2017) are also moderate in their risk of bias. Both studies employ cross-sectional designs, which are limited in their ability to establish causality and may be prone to measurement bias when interpreting cephalometric data [115,117]. On the other hand, studies like Peng et al. (2024) and Tauheed et al. (2019), although cross-sectional, show a relatively **lower risk of bias** due to larger sample sizes and well-defined statistical methodologies that reduce the chance of confounding factors influencing the results [26,119]. Longitudinal studies, such as Efendiyeva et al. (2014) and McGuinness N.J. (2006), generally present a lower risk of bias, with Efendiyeva et al. (2014) showing consistent results over time in patients undergoing treatment for Class III malocclusion [24,76]. However, even these studies face some concerns, especially due to their smaller sample sizes and the long follow-up periods that could introduce other biases, like participant dropout or changes in treatment protocols. In conclusion, while many studies utilize rigorous methodologies, they are still susceptible to varying degrees of bias, such as measurement bias, selection bias, and confounding bias. The graphical representation in Figure 4 further illustrates the varying levels of risk across the studies, showing that while some studies have a low risk, most carry moderate to high concerns that must be considered when interpreting their results.

## 4. Discussion

The relationship between craniocervical posture and Class II and III malocclusions has been extensively investigated in the literature, with several studies confirming significant differences in cervical morphology and orientation depending on the type of malocclusion.

In particular, several studies have shown that subjects with Class II malocclusion tend to present a greater cervical curvature, often associated with an anterior tilt of the skull and an increase in cervical lordosis. This can be interpreted as a biomechanical adaptation to maintain postural balance and respiratory function.

Solow and Tallgren suggest that a more extended craniocervical posture, typical of subjects with Class II, may influence craniofacial growth, supporting the hypothesis of a postural compensation mechanism. On the other hand, in patients with Class III malocclusion, a lesser cervical curvature or even a more straight cervical spine was found. This could be due to a posterior tilt of the head to compensate for the excessive mandibular projection [120].

Tecco et al. showed that subjects with Class III show a reduction in cervical lordosis, probably as a compensatory strategy for mandibular protrusion. These findings support the idea that cervical morphology is not only an element associated with malocclusion but also an integral part of a complex system of biomechanical adaptations, involving the craniocervical musculature, posture and skeletal growth [121].

In conclusion, cervical curvature in Classes II and III should not be considered only as descriptive data but interpreted in the context of postural and functional compensatory mechanisms, consistently with what has been stated by various authors.

However, to better understand these findings, it is essential to consider the biomechanical and neuromuscular mechanisms that may explain this relationship. One model that may explain the link between craniocervical posture and malocclusion is the soft tissue stretching theory. According to this hypothesis, the muscular and ligamentous structures connecting the skull to the cervical spine exert forces that can influence the position of the mandible and, consequently, dental occlusion. For instance, an abnormal head inclination can alter the tension of the supra- and infrahyoid muscles, affecting mandibular positioning.

Several studies have demonstrated that a forward head posture is associated with changes in the electromyographic activity of both masticatory and cervical muscles, suggesting that postural balance may impact mandibular dynamics [122]. Another mechanism that may explain the relationship between craniocervical posture and malocclusion is airway adaptation. Some studies have hypothesized that an altered head posture could be an adaptive response to upper airway restrictions, as seen in patients with adenotonsillar hypertrophy or obstructive sleep apnea [123].

When airway patency is compromised, the body attempts to compensate by tilting forward to facilitate airflow. This postural shift can have cascading effects on mandibular positioning, occlusal relationships, and, over time, craniofacial morphology. It has been observed that patients with abnormal head posture often exhibit malocclusive tendencies such as open bite or skeletal Class II patterns [124].

### 4.1. Craniocervical Posture Variations Across Skeletal Classes

Several studies have investigated the relationship between sagittal skeletal malocclusions and craniocervical posture, yielding partially contrasting results but collectively supporting the existence of posture-related adaptations in different malocclusion groups. Bernal et al. examined the craniocervical posture of children aged 6–11 years and found no statistically significant differences between sagittal skeletal classes. However, Class III individuals showed a tendency toward increased craniocervical flexion. Notably, 66.3% of the sample exhibited rectified lordotic curvature, with Class II subjects demonstrating increased NSL-Ver, NL-Ver, and ML-Ver values. These findings align with previous studies that suggest variations in cervical posture among malocclusion groups but highlight the need for longitudinal research to establish causality [118].

Conversely, Hosseinzadeh et al. 2011 reported a significant positive correlation between cervical column curvature and sagittal jaw relationships, particularly in Class II patients, where OPT/HOR correlated significantly with ANB (*p* < 0.05) and Wits appraisal (*p* < 0.01). The study introduced the Modified Cervical Angle (MCA), which demonstrated clinical reliability, showing the highest values in Class I individuals. These results support previous research indicating increased cervical curvature in Class II patients [113].

Similarly, Qadir and Mushtaq et al. observed significant differences in the craniometrical angle (SN-Ver) across malocclusion classes. MCA was significantly higher in Class II (6.7°) compared to Class I (3.98°, *p* = 0.0009) and Class III (5.75°, *p* = 0.0268). Class III individuals exhibited the highest OPT-Hor values (95.4°), although statistical significance was observed only in specific comparisons. Superior and inferior intervertebral spaces were significantly larger in Class III compared to Class I (*p* = 0.0487 and *p* = 0.0490, respectively), indicating a more posterior head position in Class I subjects. While these findings suggest that Class II malocclusion is associated with increased cervical curvature, the authors emphasized the necessity of further research considering the influences of age and sex [117].

The influence of head posture in craniofacial development was also emphasized in the study by Kim, Sarauw, and Sonnesen, who investigated children and adolescents with anterior open bite. While no significant differences in cervical vertebral anomalies were found between skeletal and dentoalveolar open-bite groups, a more extended head posture was observed in patients with skeletal open bite. This extended posture was strongly associated with craniofacial characteristics such as increased cranial base angle, greater vertical facial height, and jaw retrognathia. The study suggests a potential respiratory component underlying these patterns and calls for further research into the role of airway function in craniofacial development [116].

In line with this, the study by Arntsen and Sonnesen explored the relationship between cervical vertebral anomalies and head posture in children with Class II malocclusion and pronounced maxillary overjet (>6 mm). The study, involving over 200 pre-orthodontic patients, found a significantly higher prevalence of cervical spine anomalies—such as C2-C3 fusion, block fusion, and occipitalization—in the skeletal overjet group compared to the dentoalveolar group. These anomalies were associated with more pronounced sagittal discrepancies, jaw retrognathia, and an extended head posture. The findings suggest that such vertebral anomalies may be linked to deviations in craniofacial growth and reinforce the importance of evaluating cervical spine morphology in orthodontic diagnosis and treatment planning [114].

### 4.2. Natural Head Position and Skeletal Malocclusions

Several studies have explored the intricate relationship between craniocervical posture and sagittal skeletal malocclusions, highlighting how skeletal discrepancies may influence head and cervical spine positioning through postural compensations. Hedayati et al. (2013) investigated natural head posture (NHP) in adolescents aged 15–18 years and reported significant differences in cranial angles (PNS-ANS/Ver and SN/Ver) among skeletal classes. Specifically, individuals with Class III malocclusion showed a more forward head posture and a significantly smaller SN/CVT angle compared to Class I, suggesting a tendency to tuck the chin inward, possibly as a compensatory mechanism. However, no statistically significant differences were found in cervical posture angles between groups, although a trend toward a straighter cervical column was noted in Class III patients. These results suggest that adjustments in head posture may serve to compensate for underlying skeletal imbalances, particularly in Class III cases, with potential implications for orthodontic and orthognathic treatment planning [115].

The longitudinal effects of orthodontic intervention on craniocervical posture were explored by McGuinness and McDonald (2006), who assessed changes in head posture following rapid maxillary expansion (RME). While no immediate post-treatment changes were observed, significant improvements in craniocervical posture were detected one year after RME, including reductions in NSL/VER, OPT/HOR, and CVT/HOR angles. These changes likely reflect improved nasal breathing and postural adaptation over time, supporting the theory that airway function and respiratory patterns can influence head and cervical alignment. This underscores the importance of considering functional factors, such as breathing mode, in the evaluation of craniofacial development [24].

### 4.3. Cervical Curvature and Skeletal Classification

A more direct correlation between cervical curvature and skeletal classification was reported by Tauheed et al. (2019). Their study found statistically significant differences in cervical curvature (OPT/CVT) across skeletal classes, with Class III individuals exhibiting a straighter cervical spine. Additionally, Class II patients demonstrated more forward-inclined cervical postures, as indicated by lower cervicohorizontal angles. Although some findings were modest, they reinforce the idea that sagittal skeletal discrepancies are associated with adaptations in cervical posture. Nonetheless, the cross-sectional design of the study limits causal interpretations and highlights the need for longitudinal research [119].

Expanding on this, Peng et al. (2024) analyzed craniocervical posture across different stages of growth using the Cervical Vertebral Maturation (CVM) method. Their results showed significant associations between craniofacial morphology and head/neck posture during peak and post-peak growth phases. Notably, Class II patients consistently presented higher values for craniocervical angles, indicative of an extended head posture, while Class III patients displayed lower values and greater cervical inclination. These findings suggest that postural adaptations may vary throughout development and are influenced by both skeletal class and growth stage, further supporting the relevance of timing in orthodontic diagnosis and intervention [26].

The observation study by Lippold et al. investigated the possible correlation between sagittal jaw position and whole-body posture in adults. Although some links between jaw position and craniocervical tilt have been described in the literature, little research has explored the relationship with whole-body posture using validated orthopedic methods. For this reason, the authors examined 84 healthy adults who were divided into three groups based on dental overjet: Class I (normal), Class II (increased) and Class III (re-verse). Posture was analyzed using rasterstereography, a three-dimensional technique that allows the shape of the back to be accurately measured without the use of radiation. Parameters evaluated included upper thoracic tilt, kyphotic angle, lordotic angle and pelvic tilt.

The results showed no significant differences between the overjet groups for any of the postural parameters analyzed. However, comparing men and women, there were significant differences in lordotic angle and pelvic tilt [112].

### 4.4. Hyoid Bone Position

In addition to skeletal and spinal relationships, the position of the hyoid bone has been investigated as a potential indicator of postural and functional adaptations. Alexa et al. (2021) demonstrated significant differences in hyoid and cervical posture among skeletal classes, with Class II patients showing the most posterior mandibular and hyoid positioning, along with lower OPT/HOR angles suggestive of increased cervical kyphosis. Conversely, Class III individuals presented with a more anterior mandibular and hyoid position, along with signs of cervical lordosis. Hyoid bone positioning also varied according to vertical growth patterns, with hyperdivergent individuals showing greater vertical hyoid displacement. These findings underscore the complex interplay between mandibular position, cervical alignment, and soft tissue adaptation, all of which may influence both airway function and orthodontic planning [9].

Meanwhile, the comparative study by Efendiyeva et al. examines the long-term effects of bimaxillary orthognathic surgery on pharyngeal airspace, hyoid bone position and craniocervical posture in patients with Class III malocclusion. The study involved 26 adult Class III patients treated with bimaxillary surgery. Cephalometric radiographs were collected at six different time points: before treatment (T1), before surgery (T2), 5 months later (T3), 1.4 years later (T4), 3 years later (T5) and 5 years after surgery (T6). Changes in craniocervical posture, hyoid bone positioning and pharyngeal parameters were analyzed.

What emerged was that the narrowing of the pharyngeal airway observed in the postoperative period resolves in the long term, showing physiological adaptation; the position of the hyoid bone returns to normal over time. No significant effects on craniocervical posture were shown, and bimaxillary surgery does not have a negative impact on the airway in the long term; in fact, it may promote its widening [76].

### 4.5. Interpretation and Significance of Findings

The results of this study underscore a correlation between craniocervical posture and malocclusion, suggesting a complex interplay between the musculoskeletal system and occlusal structures. These findings align with previous research indicating that postural adaptations may serve as compensatory mechanisms in response to skeletal discrepancies.

From a biomechanical perspective, the observed differences in cervical curvature and head posture across malocclusion classes could be interpreted as adaptations to maintain postural stability and optimize respiratory function. For instance, the greater cervical curvature and anterior head tilt often reported in Class II patients may represent a compensatory response to maxillary retrusion and airway obstruction, as suggested by the airway adaptation hypothesis. Conversely, the more upright cervical posture observed in Class III malocclusions may reflect a posterior head tilt to counteract excessive mandibular projection. While some studies have reported strong associations between sagittal skeletal discrepancies and craniocervical posture, others have failed to identify significant differences. This inconsistency may stem from methodological heterogeneity, including variations in sample demographics, imaging techniques, and classification criteria. Standardizing these methodologies in future research will be critical to improving comparability and drawing more definitive conclusions.

### 4.6. Clinical and Research Implications

What is for sure is the fact that the methodological heterogeneity across studies is a significant limitation in interpreting results. Differences in measurement techniques, sample age, and diagnostic criteria introduce variability in findings. For example, while some studies use lateral cephalometry, others rely on three-dimensional assessments, leading to inconsistencies in reported cervical curvature values. Future research should adopt uniform assessment criteria to enhance comparability.

Craniocervical posture assessment should be integrated into orthodontic diagnosis and treatment planning: there is a need for a multidisciplinary approach to orthodontic diagnosis and treatment planning. Integrating craniocervical posture assessments into orthodontic evaluations could enhance therapeutic strategies, particularly in cases where postural imbalances contribute to malocclusion development or treatment stability. Collaboration between orthodontists, physiotherapists, and osteopaths may provide a more comprehensive framework for managing these complex interactions. Furthermore, future research should investigate whether targeted postural interventions, such as physiotherapy or airway management, could influence occlusal morphology and craniofacial growth. Advanced imaging techniques, including 3D motion analysis and functional magnetic resonance imaging, may provide deeper insights into the neuromuscular mechanisms underlying these relationships. Longitudinal studies are particularly warranted to establish causality between craniocervical posture and malocclusion and to evaluate the long-term impact of postural interventions on orthodontic outcomes.

In conclusion, rather than being a passive consequence of skeletal discrepancies, craniocervical posture appears to be an active component of a complex system of biomechanical adaptations. Understanding these interconnections will be essential for refining both diagnostic and therapeutic approaches in orthodontics and musculoskeletal medicine.

## 5. Limitations

This systematic review is subject to several limitations. First, the included studies exhibit methodological heterogeneity, with variations in sample demographics, measurement techniques, and classification criteria, making direct comparisons challenging. Second, most studies analyzed are cross-sectional, preventing causal inferences regarding the relationship between sagittal malocclusions and craniocervical posture. Third, individual factors such as age, sex, and compensatory neuromuscular adaptations may influence postural changes, yet they are not consistently accounted for across studies. Additionally, the absence of standardized protocols for assessing craniocervical posture limits the reproducibility and comparability of findings.

Another important limitation is the potential for publication bias, as studies with significant results are more likely to be published than those with negative or inconclusive findings. Furthermore, language restrictions may have introduced selection bias, as we only included studies published in English, potentially excluding relevant findings from non-English sources.

To address these limitations, future research should prioritize longitudinal designs with larger and more diverse samples to improve the generalizability of findings. Standardized measurement methods should be implemented to ensure consistency across studies, particularly in the evaluation of craniocervical posture. Moreover, controlling for confounding variables, such as growth patterns, airway function, and neuromuscular adaptations, is crucial for a more accurate interpretation of postural changes in malocclusion patients.

From a methodological perspective, the integration of three-dimensional imaging, dynamic postural assessments, and biomechanical modeling could provide deeper insights into the interplay between skeletal malocclusions and postural dynamics. Additionally, future studies should explore specific population subgroups, such as individuals undergoing orthodontic or orthopedic treatment, to assess how therapeutic interventions influence craniocervical posture over time.

By addressing these aspects, future research can refine our understanding of the complex interactions between malocclusion and posture, ultimately improving clinical management strategies.

## 6. Conclusions

The relationship between craniocervical posture and malocclusion extends beyond descriptive data and should be interpreted within a broader biomechanical and functional framework. By integrating findings across studies and considering compensatory mechanisms, a more comprehensive understanding of this interaction can be achieved. Addressing methodological heterogeneity, adopting standardized evaluation methods, and exploring clinical applications will enhance the relevance of future research in this field.

This systematic review confirms a pattern of association between sagittal skeletal malocclusions and craniocervical posture. Specifically, Class II malocclusions are frequently linked to an increased cervical curvature and a more extended head posture, while Class III individuals often present a straighter cervical spine and a forward head position. These postural adaptations may reflect compensatory mechanisms aimed at preserving functional balance.

Clinically, these findings highlight the relevance of assessing craniocervical posture during orthodontic evaluation and treatment planning, especially in patients with significant sagittal discrepancies.

Future studies should prioritize longitudinal designs and adopt standardized protocols for posture assessment—including consistent head positioning, cephalometric landmarks, and age stratification—to better define the directionality and clinical impact of these relationships. Furthermore, exploring the integration of postural evaluation into orthodontic diagnostic frameworks could lead to more comprehensive and individualized treatment approaches.

## Figures and Tables

**Table 1:** PICOS table.

Criterion	Description
Population (P)	Individuals with skeletal Class I, II, or III malocclusions, diagnosed through cephalometric analysis.
Exposure (E)	Craniocervical posture assessed through cephalometric measurements and cervical curvature analysis.
Comparison (C)	Differences in postural parameters between individuals with different skeletal classifications or comparisons with normocclusal subjects.
Outcome (O)	Association between craniocervical cephalometric parameters (e.g., cervical angles and head position) and sagittal skeletal classification.
Study Design (S)	Observational studies (cross-sectional, case–control, and longitudinal) evaluating craniocervical posture in relation to sagittal malocclusion.

**Table 2:** Descriptive summary of item selection.

Author and Year	Study Type	Materials and Methods	Aim of the Study	Key findings
Mcguinness N.J. (2006) [24]	Longitudinal Observational study	43 patients with uni- or bilateral crossbite	To evaluate the effects of rapid maxillary expansion (RME) on natural head posture (NHP) by analyzing changes immediately after treatment and one year later.	No significant differences in cervical posture among groups (*p* > 0.05).
Lippold C. (2006) [112]	Observational cross-sectional study	84 patients divided by OVJ in Class I(18), class II (38), Class III (28)	To analyze posture through rasterstereography, which determined upper thoracic tilt, kyphotic angle, lordotic angle, and pelvic tilt.	There is no association between sagittal jaw position and body posture in adults.
Hosseinzadeh Nik T et al. (2011) [113]	Cross-sectional observational study	100 pre-treatment lateral cephalograms (56 females, 44 males; mean age: 13.49 ± 5.53 years). Classification: Angle’s malocclusion (Class I, II, III). Measurement: Modified constructed CVT/HOR and OPT/HOR angles. Statistical analysis: correlation tests.	To evaluate cervical posture and curvature using modified constructed angles and their relationship with sagittal jaw position	Class II showed increased cervical curvature.
Arntsen et al. (2011) [114]	Observational cross-sectional study.	213 patients divided into a skeletal overjet group and a dentoalveolar overjet group	To examine the morphology of the cervical vertebral column in preorthodontic children with Class II malocclusion and a horizontal maxillary overjet of more than 6 mm.	Class II was associated with extended head posture.
Hedayati et al. (2013) [115]	Retrospective observational study	102 lateral cephalometric radiographs (age 15–18 years) divided into Class I (n = 32), Class II (n = 40), Class III (n = 30). Measured 11 craniofacial and cervical landmarks. Statistical analysis: ANOVA and post hoc tests.	To evaluate natural head position (NHP) differences among Class I, II, and III skeletal malocclusions.	Class III had a more forward head posture.
Efendiyeva et al. (2014) [76]	Longitudinal Observational study	26 patients with III class malocclusion	To evaluate the effects of bimaxillary orthognathic surgery on pharyngeal airway space, hyoid bone position, and craniocervical posture in Class III patients, both in the short- and long-term.	No significant difference in craniocervical angulation.
Kim P. (2014) [116]	Observational cross-sectional study	111 patients divided into a skeletal open-bite group and a dentoalveolar open-bite group	Cervical vertebral column morphology and head posture in preorthodontic patients with anterior open bite.	Head posture was significantly more extended in the skeletal open-bite group.
Qadir and Mushtaq (2017) [117]	Cross-sectional observational study	90 subjects (43 males, 47 females, aged 15–35 years). Lateral cephalograms analyzed for cervical curvature and sagittal jaw relationship. Statistical tests included intergroup comparisons.	To examine the relationship between cervical column curvature and sagittal jaw position.	Lordotic curvature was not significantly different among groups.
Bernal et al. (2017) [118]	Cross-sectional descriptive study	107 children (55 girls, 52 boys), aged 6–11 years. Lateral skull radiographs analyzed with NEMOTEC software. 13 variables measured: age, gender, ANB angle, and 10 craniocervical posture indicators. Intra-class correlation coefficients ranged 0.945–0.996	To evaluate the craniocervical posture of children and its relationship with sagittal skeletal classification.	Class III had a tendency for greater craniocervical flexion.
Tauheed et al. (2019) [119]	Cross-sectional study	63 subjects (11–22 years), cephalometric radiographs, ANB classification, cervical posture measured using OPT/HOR, CVT/HOR, and OPT/CVT	To determine cervical posture in different skeletal sagittal malocclusions and assess its correlation with skeletal relationships	Forward head posture was more prevalent in Class II subjects (*p* < 0.05).
Alexa et al., (2022) [9]	Observational, retrospective	45 lateral cephalograms (patients aged 25–30); ANB and FMA angles used for classification; cephalometric analysis with AudaxCeph^®^ software; statistical tests: ANOVA, Duncan test	Investigate relationships between cervical posture, head position, and hyoid bone position in orthodontic patients with different skeletal patterns	Class II patients had increased cervical kyphosis, class III had increased cervical lordosis.
Peng et al., (2024) [26]	Cross-sectional study	150 lateral cephalometric radiographs (75 males, 75 females, aged 7–18). Classified into growth stages (CVM) and skeletal classes (ANB angle). Statistical analysis using SPSS 26.0, Pearson’s correlation, ANOVA.	Investigate the correlation between craniofacial morphology and craniocervical posture in patients with sagittal skeletal malocclusion across different growth stages.	Class III individuals exhibited a straighter cervical spine compared to Class I and II.

## Abbreviations

Abbreviation	Full Form
ANB	A point-Nasion-B point (used to classify the sagittal relationship of the dental arches)
CCA	Cervical Column Angle
CMDs	Craniomandibular Disorders
CVT/HOR	Cervical Vertebrae Tangent/Horizontal
FMA	Frankfort Mandibular Plane Angle
MCA	Modified Cervical Angle
ML-Ver	Mandibular Line/Vertical
NHP	Natural Head Position
NL-Ver	Nasal Line/Vertical
NOS	Newcastle-Ottawa Scale
NSL/VER	Nasion-Sella Line/Vertical
OPT/CVT	Optic Plane to Cervical Vertebrae Tangent
OPT/HOR	Optic Plane/Horizontal
OVJ	Overjet
RME	Rapid Maxillary Expansion
SN-Ver	Sella-Nasion to Vertical Plane
SN/CVT	Sella-Nasion to Cervical Vertebrae Tangent
TENS	Transcutaneous Electrical Nerve Stimulation
